# Supplementary material for: Content-rich biological network constructed by mining PubMed abstracts
Source: BMC Bioinformatics. 2004 Oct 8;5:147. doi: 10.1186/1471-2105-5-147 (PMC528731; doi:10.1186/1471-2105-5-147)
Supplement: Additional File 2 — The original results of the above study (non-essential files are deleted to keep the file size under the limit set by BMC bioinformatics). [file 1471-2105-5-147-S2.bz2 › chilibotAdditionalFile2/dip30/394ID8702666E9948/html/EGF_EGFR.html]

 


 **EGF** and **EGFR** 
  
Found 3495 abstracts in PubMed, retrieved 30.  
 

 What does Google say? 
 PDF only 
| .edu only 

---

**Interactive relationship** (e.g. stimulation, inhibition, etc)

**Inhibitory relationship**- Intracellular expression of the aptamer encoding gene construct KDI1 or introduction of bacterially expressed KDI1 via a protein transduction domain into  **EGFR**  expressing cells results in KDI1  **EGF**  receptor complex formation, a slower proliferation and reduced soft agar colony formation.  Ref: 12842895 J Biol Chem< MedlineTA>J Biol Chem,
- Co treating PC 3 cells with quercetin significantly attenuated  **EGF**  and TGF alpha induced growth and phosphorylation of ErbB  [ **EGFR** ]  2, ErbB  [ **EGFR** ]  3, c Raf, MAPK kinase 1 2 MEK1 2, MAPK, Elk 1 and Akt 1.  Ref: 12888923 Int J Oncol, 2003
**Neutral relationship**- BTC induced phosphorylation of all four  **EGF**  receptors present on HTASMCs ErbB1  [ **EGFR** ] , ErbB2, ErbB3, and ErbB4.  Ref: 12869389 Circ ResCirc Res, 2003
- 1 Association of Grb2 to  **EGFR**  in BxPC 3 induced by  **EGF**  in the presence of Erbitux indicates an alternate pathway of Ras MAPK activation, which may be related with the tumor resistance to treatment.  Ref: 12850474 J Surg Res, 2003
- In undigested, matrix laden fibroblasts, HB  **EGF**  significantly down regulates elastin mRNA via activation of epidermal growth factor receptor  [ **EGFR** ]   **EGF**  R.  Ref: 12882762 Am J Physiol Lung Cell Mol PhysiolAm J Physiol Lung Cell Mol Physiol, 2003
- This effort identified ErbB4, one of the  **EGF**  heregulin receptors, and Grb7, an adapter protein associated with ErbB4 ErbB  [ **EGFR** ] , epidermal growth factor receptor  [ **EGFR** ]  family protein.  Ref: 12853971 Oncogene, 2003
- To test a functional role for HB  **EGF**  in ET 1 signaling, we showed that exogenous HB  **EGF**  stimulated phosphorylation of ErbB1  [ **EGFR** ]  and growth of mesangial cells.  Ref: 12853287 Am J Physiol Cell Physiol< MedlineTA>Am J Physiol Cell Physiol, 2003

**Non-interactive relationship** (e.g. studied together, co-existance, homology, etc.)

- Plasma membrane phospholipid scramblase 1 promotes  **EGF**  dependent activation of c Src through the epidermal growth factor receptor  [ **EGFR** ] .  Ref: 12871937 J Biol ChemJ Biol Chem, 2003
- Further studies are necessary to charify whether  **EGF**   **EGFR**  play a significant role in the pathogenesis of CAG.  Ref: 12881871 Zhejiang Da Xue Xue Bao Yi Xue BanZhejiang Da Xue Xue Bao Yi Xue Ban, 2003
- Immunoblots showed that Grb2 was co immunoprecipitated with  **EGFR**  after  **EGF**  stimulation.  Ref: 12850474 J Surg Res, 2003
- Co immunoprecipitation and immunolocalization demonstrated that  **EGF**  co localized with PKCalpha and  **EGFR**  PMA did not abrogate  **EGFR**  transactivation.  Ref: 12878187 Biochem Biophys Res Commun, 2003
- To determine the utility of this approach, we simulated the differential activation of the  **EGFR**  by two of its ligands, epidermal growth factor  **EGF**  and transforming growth factor alpha TGF alpha.  Ref: 12885624 Biophys J, 2003
- Various chimeras of the ErbB1  [ **EGFR** ]  specific ligands  **EGF**  and TGFalpha display an enlarged repertoire as activators of ErbB2.ErbB3 heterodimers.  Ref: 12869572 J Biol ChemJ Biol Chem, 2003
- As expression and activation levels of the epidermal growth factor receptor  [ **EGFR** ]   **EGFR**  predict the metastatic potential of human colorectal cancers, the purpose of this study was to establish whether the  **EGF**  receptor tyrosine kinase  **EGFR**  TK contributes to cellular invasion induced by TFFs in kidney and colonic cancer cells.  Ref: 12881705 Oncogene, 2003
- The epidermal growth factor  **EGF**  family of tyrosine kinase receptors erbB  [ **EGFR** ]  receptors are expressed at high levels in a wide variety of human cancers and have been associated with various features of advanced disease and poor prognosis.  Ref: 12857977 Immunol Res, 2003
- Among the best studied growth factor receptors are the two members of  **EGF**  receptor familiy  **EGFr**  ErbB 1  [ **EGFR** ] , and Her2 neu ErbB  [ **EGFR** ]  2.  Ref: 12850530 Crit Rev Oncol Hematol, 2003
- To evaluate heterodimer formation and additional transactivation by  **EGF** , we investigated the roles of the four extracellular subdomains of p185 c neu and the  **EGFR** .  Ref: 12867596 Proc Natl Acad Sci U S AProc Natl Acad Sci U S A, 2003
- RESULTS The average  **EGF**  level 0.149 0.020 μg L and 80% positive rate of  **EGFR**  expression observed in CAG group were significantly higher than those in control group 0.043 0.003 μg L and 0%, P< ; 0.01.  Ref: 12881871 Zhejiang Da Xue Xue Bao Yi Xue BanZhejiang Da Xue Xue Bao Yi Xue Ban, 2003
- Phosphorylated  **EGFR**  p185 c neu heterodimeric forms were detected in the absence of  **EGF** , but the species became highly phosphorylated after  **EGF**  stimulation.  Ref: 12867596 Proc Natl Acad Sci U S AProc Natl Acad Sci U S A, 2003
- The ability of CIN85 to cluster Cbl was important for ligand induced stabilization of CIN85 Cbl  **EGFR**  complexes, as well as for  **EGF**  receptors degradation in the lysosome.  Ref: 12874286 J Biol ChemJ Biol Chem, 2003
- Cell lysates were harvested after cells were stimulated with  **EGF** , FGF, or IGF 1 respectively, and  **EGFR**  was immunoprecipitated using Erbitux.  Ref: 12850474 J Surg Res, 2003
- The recombinant protein could produce antibodies to recognize extracellular domain and full length of mouse  **EGFR** , and form homodimer in the presence of  **EGF**  detected by western blot analysis.  Ref: 12856594 Sheng Wu Yi Xue Gong Cheng Xue Za Zhi, 2003
- With  **EGF**  liposomes we present a pegylated ligand liposome delivery vehicle, containing water soluble boronated phenanthridine, WSP1, or water soluble boronated acridine, WSA1, for  **EGFR**  targeting.  Ref: 12851696 Int J Oncol, 2003
- Similarly,  **EGFR**  TK inhibition by ZD1839 reversed the invasive phenotype promoted by the constitutively activated form of the  **EGFR**  EGFRvIII and the  **EGFR**  agonists transforming growth factor alpha TGFalpha, amphiregulin and  **EGF** .  Ref: 12881705 Oncogene, 2003
- OBJECTIVE To investigate the epidermal growth factor  **EGF**  and epidermal growth factor receptor  [ **EGFR** ]   **EGFR**  expression in atrophic gastritis in rats to explore the relationship between  **EGF**  and chronic atrophic gastritis CAG.  Ref: 12881871 Zhejiang Da Xue Xue Bao Yi Xue BanZhejiang Da Xue Xue Bao Yi Xue Ban, 2003
- Coimmunoprecipitation experiments also indicated that Grb7 and NIK could be simultaneously recruited into signaling complexes of all known  **EGF**  heregulin receptors, including  **EGFR** , ErbB2, ErbB3, and ErbB4.  Ref: 12853971 Oncogene, 2003
- The content of urinary bladder cancer antigen UBC, tissue polypeptide specific antigen TPS, nuclear matrix protein 22 NMP22, and the expression of LewisY carbohydrate antigens and of epidermal growth factor receptor  [ **EGFR** ]   **EGF**  R in bladder tumor tissues were determined.  Ref: 12888914 Int J Oncol, 2003
- Detachment induced expression of Bim requires a lack of beta 1 integrin engagement, downregulation of  **EGF**  receptor  **EGFR**  expression and inhibition of Erk signalling.  Ref: 12844146 Nat Cell Biol< MedlineTA>Nat Cell Biol, 2003
- Structural analysis of an  **EGF**  TGFalpha chimera with unique ErbB  [ **EGFR** ]  binding specificity.  Ref: 12869572 J Biol ChemJ Biol Chem, 2003
- CONCLUSION The study demonstrates an association of high levels of  **EGF**  and positive  **EGFR**  expression in CAG rats.  Ref: 12881871 Zhejiang Da Xue Xue Bao Yi Xue BanZhejiang Da Xue Xue Bao Yi Xue Ban, 2003
- Our simulations predict that when  **EGFR**  is activated with TGF alpha, receptor activation is biased toward the cell surface whereas  **EGF**  produces a signaling bias toward the endosomal compartment.  Ref: 12885624 Biophys J, 2003
- METHODS The serum  **EGF**  and gastric mucosal,  **EGFR**  level were measured in 20 rats with CAG and 20 normal controls.  Ref: 12881871 Zhejiang Da Xue Xue Bao Yi Xue BanZhejiang Da Xue Xue Bao Yi Xue Ban, 2003
- Internalization rates of  **EGFR**  induced by Erbitux on these cell lines were determined using 125I  **EGF**  binding assay after removal of Erbitux by acidic wash.  Ref: 12850474 J Surg Res, 2003
- Inhibition of ErbB  [ **EGFR** ]  2 and ErbB  [ **EGFR** ]  3 expression by quercetin prevents transforming growth factor alpha TGF alpha and epidermal growth factor  **EGF**  induced human PC 3 prostate cancer cell proliferation.  Ref: 12888923 Int J Oncol, 2003
- Effects of  **EGF**   **EGFR**  expression on atrophic gastritis in rats .  Ref: 12881871 Zhejiang Da Xue Xue Bao Yi Xue BanZhejiang Da Xue Xue Bao Yi Xue Ban, 2003
